# High Morphologic Plasticity of Microglia/Macrophages Following Experimental Intracerebral Hemorrhage in Rats

**DOI:** 10.3390/ijms17071181

**Published:** 2016-07-21

**Authors:** Shu-Sheng Yang, Li Lin, Yue Liu, Jie Wang, Jiang Chu, Teng Zhang, Lin-Na Ning, Yan Shi, Ying-Yan Fang, Peng Zeng, Jian-Zhi Wang, Ming-Yi Qiu, Qing Tian

**Affiliations:** 1 Department of Pathology and Pathophysiology, School of Basic Medicine; Institute for Brain Research, Huazhong University of Science and Technology, Hangkong Road 13#, Wuhan 430030, China; yangss@stmail.hbtcm.edu.cn (S.-S.Y.); linli@hbtcm.edu.cn (L.L.); chujiang@hust.edu.cn (J.C.); zhangteng012@hust.edu.cn (T.Z.); ninglinna@hust.edu.cn (L.-N.N.); shiy@hust.edu.cn (Y.S.); fyingyan@hust.edu.cn (Y.-Y.F.); zengp@hust.edu.cn (P.Z.); wangjz@mails.tongji.edu.cn (J.-Z.W.); 2 Department of Shang-Han, Clinical College of Traditional Chinese Medicine, Hubei University of Traditional Chinese Medicine, Tan-Hua-Lin Road 1, Wuhan 430061, China; 3 Laboratory of Medical Molecular and Cellular Biology, School of Basic Medicine, Hubei University of Traditional Chinese Medicine, Wuhan 430065, China; 4 Wuhan National Laboratory for Optoelectronics, Huazhong University of Science and Technology, Wuhan 430074, China; lydhr@hust.edu.cn; 5 Wuhan Institute of Physics and Mathematics, Chinese Academy of Sciences, Wuhan 430071, China; jie.wang@wipm.ac.cn

**Keywords:** microglia, intracerebral hemorrhage, interleukin-1β, interleukin-10

## Abstract

As current efforts have limited effects on the clinical outcome of intracerebral hemorrhage (ICH), the mechanisms including microglia/macrophages that involved inflammation need further investigation. Here, 0.4 units of collagenase VII were injected into the left caudate putamen (CPu) to duplicate ICH rat models. In the brains of ICH rats, microglia/macrophages, the nearest cells to the hemorrhagic center, were observed as ameboid and Prussian-blue positive. Furthermore, the ameboid microglia/macrophages were differentiation (CD) 68 and interleukin-1β (IL-1β) positive, and neither CD206 nor chitinase3-like 3 (Ym1) positive, suggesting their strong abilities of phagocytosis and secretion of IL-1β. According to the distance to the hemorrhagic center, we selected four areas—I, II, III, and IV—to analyze the morphology of microglia/macrophages. The processes decreased successively from region I to region IV. Microglia/macrophages in region IV had no processes. The processes in region I were radially distributed, however, they showed obvious directivity towards the hemorrhagic center in regions II and III. Region III had the largest density of compactly arrayed microglia/macrophages. All these in vivo results present the high morphologic plasticity of microglia/macrophages and their functions in the pathogenesis of ICHs.

## 1. Introduction

Intracerebral hemorrhages (ICHs) account for 8%–15% of all strokes in high-income countries [1] and a higher percentage in Asia [2]. Genetic variants of apolipoprotein E, ethnic differences and lifestyle factors such as smoking and alcohol consumption are major risk factors for ICH [3]. Multiple complicated factors including the initial hematoma volume, hematoma expansion during the first day, location of the hematoma, extent of brain edema, age and neurological status on admission influence the clinical outcomes [4,5]. It is important to understand molecular and cellular mechanisms underlying early brain damage after ICHs. In ICHs, the appearance of extracellular blood, resulting in the release of the hemoglobin constituents, heme and iron, triggers specific pathophysiological cascades or modifies the timing of other processes, including inflammation.

Microglia, the brain-resident macrophages [6] accounting for about 10% of the total glial population, are associated with the pathogenesis of hemorrhage injuries. In response to injuries, the resident microglia/macrophages are rapidly mobilized to the injury site and initiate inflammatory response [7], altering their morphology and phenotype to adopt a so-called activated state. Activated microglia/macrophages were believed to phagocyte the dying cells and debris and/or release some cytokines to maintain the homeostasis of the microenvironment for supporting the injured neurons [8,9]. As an active sensor and monitor in the brain, one important characteristic of microglia/macrophages is high morphologic and functional plasticity. Depending on the cytokine environment present during microglia/macrophage activation, microglia/macrophages can be categorized into two broad phenotypes [10], pro-inflammatory (M1) and anti-inflammatory (M2). The M1 phenotype upregulates phenotypic markers including cluster of differentiation (CD) 86, CD68, CD16, CD32 and the major histocompatibility complex class II (MHC-II) and releases pro-inflammatory cytokines including interleukin (IL)-1β, IL-6, tumor necrosis factor-α (TNF-α) and nitric oxide (NO) [11,12,13]. The M2 phenotype expresses chitinase 3-like 3 (Ym1), CD206, arginase 1, and anti-inflammatory cytokines, such as IL-4 and IL-10 [10,12,13].

In this research, we injected 0.4 units (U) of collagenase VII into the left caudate putamen (CPu) of three-month-old male Sprague–Dawley (SD) rats and successfully duplicated ICH rat models. By morphological techniques including immunofluorescence labeling and immunohistochemical staining, we studied the distribution, morphology and some functional characteristics of microglia/macrophages. It was observed that microglia/macrophages, not astrocytes, were the nearest cells to the hemorrhagic center, and they were ameboid. In addition, these Prussian-blue positive ameboid microglia/macrophages were IL-1β and CD68 positive. Microglia/macrophages in the hemorrhagic CPu also had high morphologic plasticity characteristics. All of these results from in vivo observations present the high morphologic plasticity of microglia/macrophages and their important roles in the pathogenic process of ICH.

## 2. Results

### 2.1. Increased and Accumulated Microglia/Macrophages and Astrocytes in the Hemorrhagic CPU of Rats

**Figure 1:**
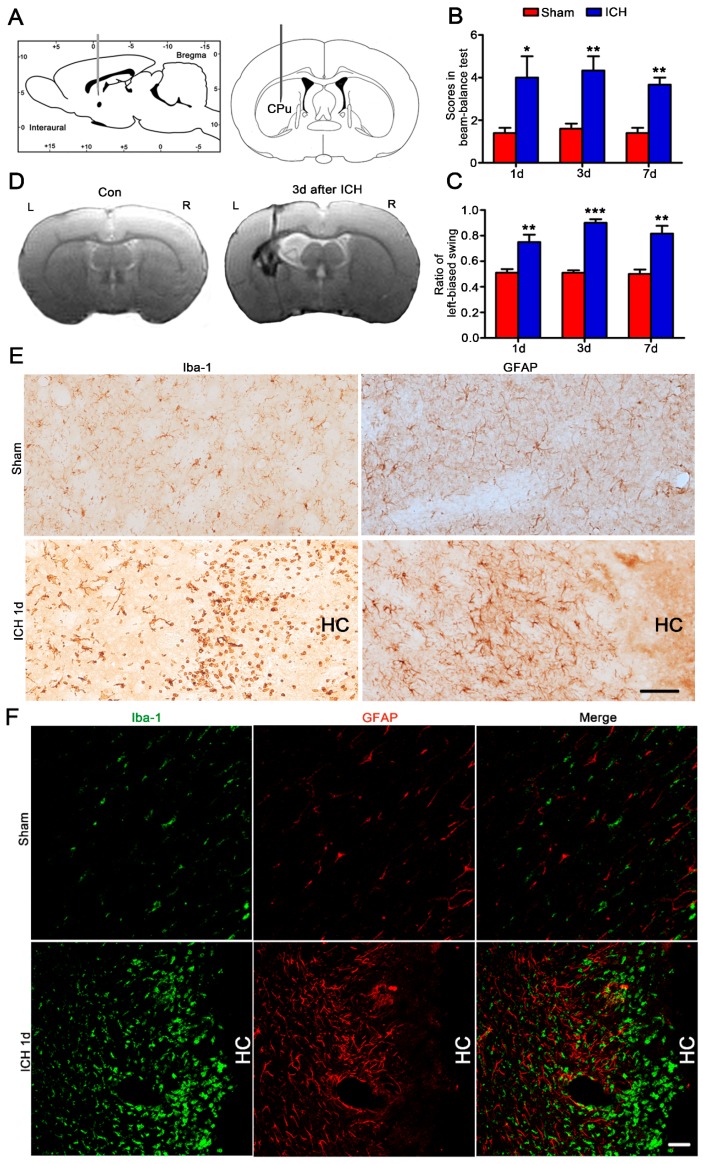
Increased and accumulated microglia/macrophages and astrocytes in the hemorrhagic caudate putamen (CPu) of rats. 0.4 U of collagenase VII (2 μL) was injected into the left CPu of three-month-old male Sprague–Dawley (SD) rats (**A**, ICH). Sham rats (Sham) received 2 μL 0.9% NaCl injection. Beam balance test (**B**, *n* = 5/group), elevated body swing test (**C**, *n* = 5/group), and brain magnetic resonance imaging (MRI) test (**D**, *n* = 4/group) were performed to evaluate the model. By immunohistochemistry staining on brain slices (20 μm), Iba-1 (marker of microglia/macrophage) positive cells and GFAP (marker of astrocyte) positive cells were shown in the collagenase VII injected CPu (**E**, Bar = 50 μm). By double-label immunofluorescence staining, microglia/macrophages (green) were found much closer to the hemorrhagic center (HC) than astrocytes (red) (**F**, Bar = 50 μm). The data were expressed as means ± standard error of the mean (SEM).* *p* < 0.05, ** *p* < 0.01, *** *p* < 0.001, vs. Sham.

As part of the experiment, 0.4 U collagenase VII was injected into the left CPu of three-month-old male SD rats as reported [14] (Figure 1A). The beam balance test and elevated body swing test were performed to evaluate the limb strength of rats at one day, three days and seven days after collagenase VII injection. In a two-minute beam balance test, ICH rats had more drop times than sham rats and they often fell down from the right side (Figure 1B). In an elevated body swing test, ICH rats showed more left-biased swings than sham rats (Figure 1C). These tests showed obvious decreased muscle strength of the right limbs in ICH rats. By the brain MRI test at three days after injection, the hemorrhagic foci were clearly showed (Figure 1D) and the volume of lesion area in the brain was about 0.058 cm^3^. These data proved that collagenase VII was successfully used to duplicate CPu hemorrhage in rats. To identify the cells around the hemorrhagic foci, we stained the brain slices of ICH rats at one day after operation with antibodies specific to Iba-1 (marker of microglia/macrophages) and GFAP (marker of astrocytes) by immunohistochemistry and immunofluorescence staining. Both microglia/macrophages and astrocytes were observed increasing and accumulating in the collagenase VII-injected CPu (Figure 1E,F). However, Iba-1 positive cells, but not GFAP positive cells, were found much closer to the hemorrhagic center (HC) by double-label immunofluorescence staining (Figure 1F). All these data indicated the important roles of these increased and accumulated microglia/macrophages in the pathological process of ICH.

### 2.2. Microglia/Macrophages Nearest to the Hemorrhagic Center Were Ameboid and Prussian Blue Positive

**Figure 2:**
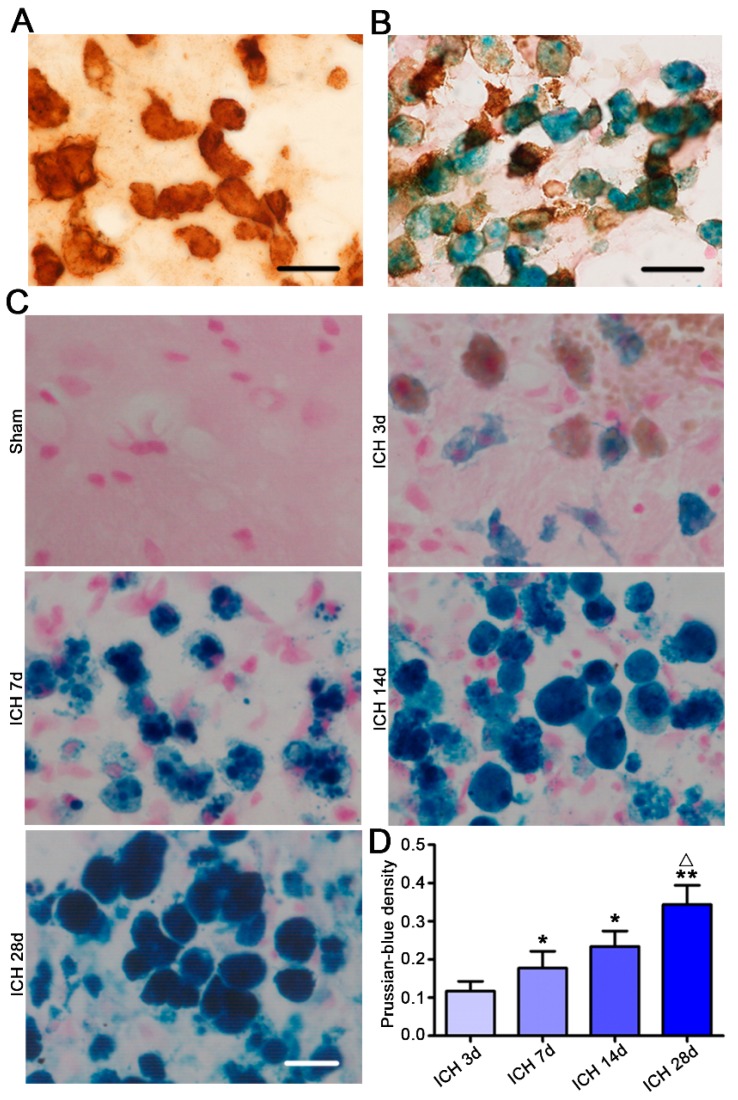
Ameboid microglia/macrophages in the hemorrhagic center presented Prussian blue positive. Brain slices (20 μm) of intracerebral hemorrhage (ICH) rats were immunohistochemically stained by Iba-1 (**A**, *n* = 5) or further double stained with Prussian blue (**B**,**C**, *n* = 5). The optical densities of Prussian-blue staining were quantitatively analyzed in **D** (*n* = 5). Microglia/macrophages in the brain slices taken at 7, 14 and 28 days had stronger blue staining than those in three-day brain slices (**C**,**D**). Bar = 25 μm. Data were expressed as means ± SEM. * *p* < 0.05, ** *p* < 0.01, vs. ICH 3 d. Δ *p* < 0.05, vs. ICH 7 d.

By immunohistochemistry, the microglia/macrophages nearest to the hemorrhagic center were observed ameboid in the brain slices of ICH rats (Figure 2A). Then, to understand the possible functions of these ameboid microglia/macrophages, we used Prussian blue staining. After the erythrocytes are phagocytized into microglia/macrophages, hemoglobin will be decomposed into hematoidin and hemosiderin by lysosomal enzyme. After Prussian blue staining hemosiderin presents blue. In Prussian blue staining kits and anti-Iba-1-marked immunohistochemistry stained brain slices, ameboid Iba-1 positive microglia/macrophages were shown Prussian blue positive (Figure 2B), suggesting their ability of phagocytizing the blood constituents into the brain and important role in the cleaning of hemorrhage. It was also shown that microglia/macrophages in the brain slices taken at 7-, 14- and 28 days had stronger blue staining than those in three-day brain slices (Figure 2C), indicating more hemosiderin included in ameboid microglia/macrophages. However, if it was because of ameboid microglia/macrophages having stronger phagocytosis ability, this needs further investigation.

### 2.3. Ameboid Microglia/Macrophages Presented CD68 and IL-1β Positive

**Figure 3:**
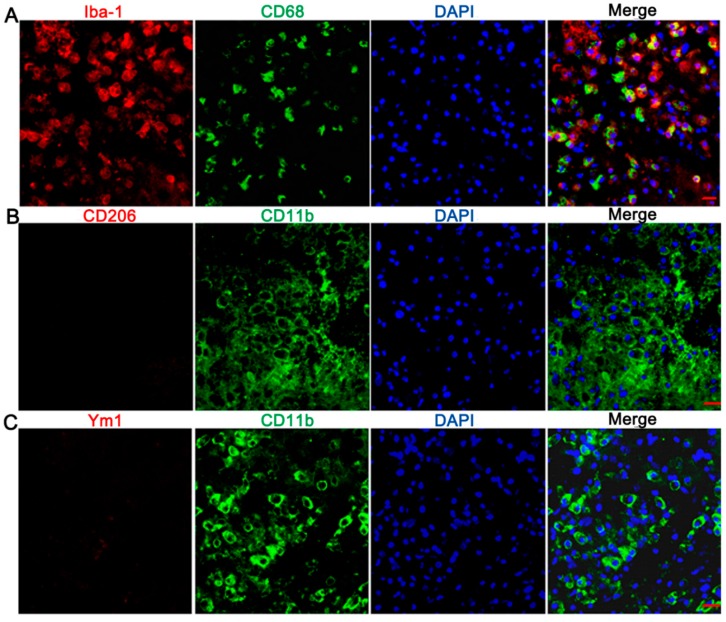
Ameboid microglia/macrophages were mainly differentiation (CD)68 positive in the hemorrhagic center. Brain slices (20 μm) were taken from ICH rats at three days after collagenase VII injection. By double-label immunofluorescence staining, ameboid Iba1 positive (red, **A**) or CD11b positive (green, **B**,**C**) microglia/macrophages in the hemorrhagic center were CD68 positive (green, **A**, *n* = 3), neither CD206 nor chitinase 3-like 3 (Ym1) positive (red, **B**,**C**) (**B** and **C**, *n* = 3). Bar = 30 μm.

**Figure 4:**
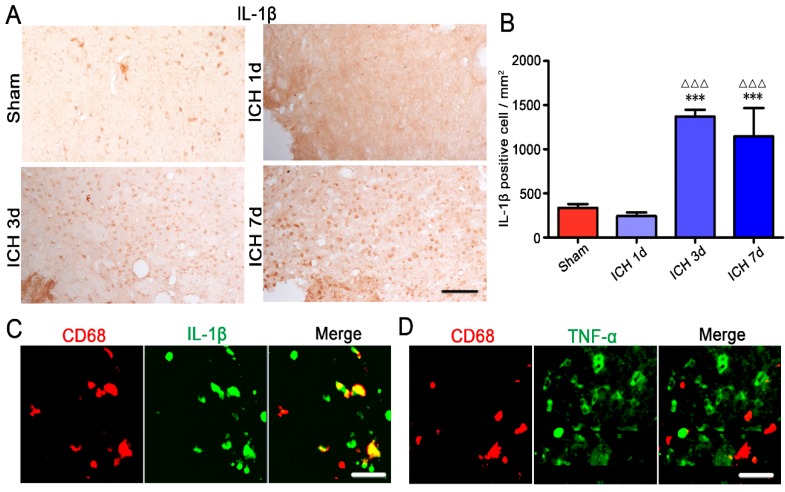
Increased IL-1β in the hemorrhagic CPu was detected at three and seven days after collagenase VII injection. IL-1β in the CPu was studied by immunohistochemistry (**A**,**B**, Bar = 100 μm) on brain slices (20 μm) at one, three, and seven days after collagenase VII injection (ICH 1 d, 3 d and 7 d, *n* = 3). Control CPu (Sham) was from 2 μL 0.9% NaCl injected rats (*n* = 3). Data were expressed as means ± SEM. *** *p* < 0.001, vs. Sham. ΔΔΔ *p* < 0.001 vs. ICH 1 d. To study the cytokine expressions of activated microglia in the hemorrhagic center, brain slices were double-stained with antibodies specific to pro-inflammatory cytokines interleukin (IL)-1β (**C**, green) ortumor necrosis factor-α (TNF-α) (**D**, green) and CD68 (red in **C**,**D**, *n* = 3). Bar = 50 μm.

Microglia/macrophages have multiple activation phenotypes [15,16]. Depending on the cytokine environment present during microglia/macrophage activation, they can be categorized into two broad phenotypes [10]. The classical M1 phenotype of microglia/macrophage produces high levels of pro-inflammatory cytokines including IL-1β, IL-6 and TNF-α, and NO, which are essential for host defense and phagocytic activity but that also induce damage to un-injured tissues [11]. In contrast, the M2 phenotype expresses anti-inflammatory cytokines, such as IL-4 and IL-10, which are beneficial for brain damage repair [10,12]. CD68, a lysosomal activity marker expressed during inflammatory processes by microglia/macrophages, is regarded as a marker of M1 microglia/macrophages [13,17,18]. Activated microglia were reported to be labeled by CD11b immunoreactivity [19], and antibodies specific to CD206 and Ym1 are used to identify M2 microglia/macrophages [20]. In this study, it was observed that the ameboid Iba-1 positive microglia in the hemorrhagic center were CD68 positive, in brain slices taken at three days (Figure 3A) and even at 7, 14 and 28 days [21] after collagenase VII injection. In addition, the ameboid CD11b positive cells were neither CD206 (Figure 3B) nor Ym1 (Figure 3C) positive. We also studied IL-1β in hemorrhagic CPu by immunohistochemistry and found IL-1β had higher levels at three to seven days after collagenase VII injection (Figure 4A,B). By double-label immunofluorescence staining, CD68 positive cells in the hemorrhagic center were found to be IL-1β positive (Figure 4C), not TNF-α positive (Figure 4D). All these data suggested that ameboid microglia/macrophages in the hemorrhagic center present the M1 phenotype and secrete IL-1β selectively. The selectivity of pro-inflammatory cytokines secretion by the M1 phenotype needs further investigation.

### 2.4. High Morphologic Plasticity of Microglia/Macrophages in Hemorrhagic CPu

**Figure 5:**
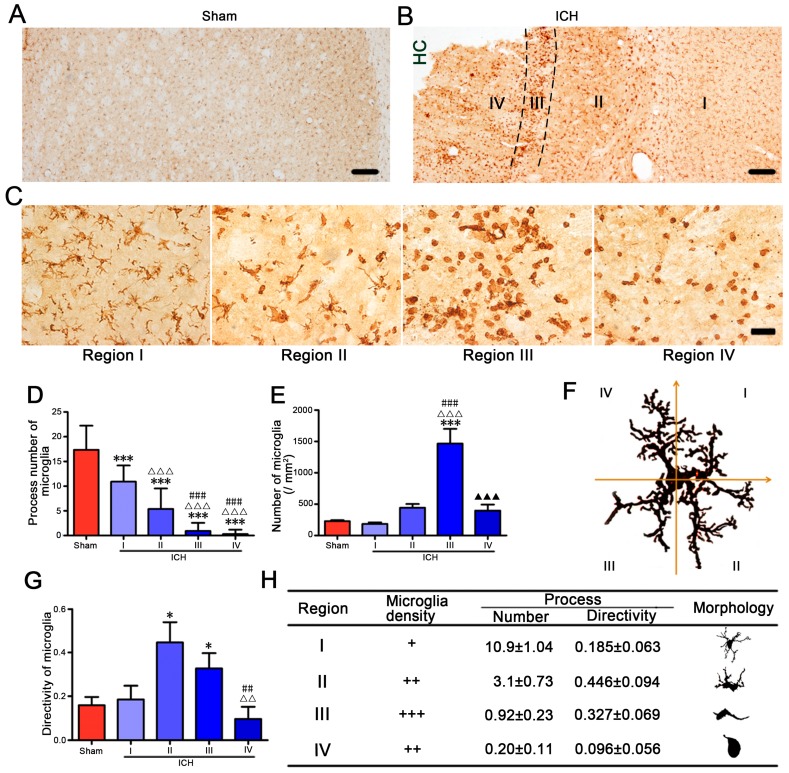
Microglia/macrophages showed high morphologic plasticity. Brain slices (20 μm) were taken three days after operation from Sham rats (**A**) and ICH rats (**B**,**C**). By immunohistochemistry, Iba-1 marked microglia/macrophages in the left CPu were observed (*n* = 3, Bar = 200 μm). According to the distance to the hemorrhagic center (HC), we selected four areas (**B**,**C**) to analyze the morphologic features of activated microglia/macrophages, and the left CPu of sham rats was used as control (Sham, **A**,**D**,**E**). The process numbers (**D**) and the densities (**E**) of microglia/macrophages were studied. To evaluate the process directivity of a microglia/macrophage, the cell body was set as the origin, and the *y*-axis was set pointing to the hemorrhagic center (**F**). The directivity was calculated as the process number in quadrants I and IV deducted the number in quadrants II and III, and the difference was divided by the total process number (**G**). The morphologic characteristics of activated microglia/macrophages after ICH was summarized in (**H**). Data were expressed as means ± SEM. * *p* < 0.05, *** *p* < 0.001, vs. Sham. ΔΔ *p* < 0.01, ΔΔΔ *p* < 0.001, vs. region I. ## *p* < 0.01, ### *p* < 0.001, vs. region II. ▲▲▲ *p* < 0.001, vs. region III.

Microglia/macrophages are sensitive to brain injury, altering their morphology and phenotype to adopt a so-called activated state in response to brain insults. In brain slices taken at one, three and seven days after operation, we observed the obviously increased microglia/macrophages in collagenase VII-injected CPu (Figure 5A,B, data taken at one and seven days after injection not shown). A band with about 120–150 μm width formed by concentrated arrayed microglia/macrophages surrounded the hemorrhagic foci (Figure 5B). According to the distance to the band, we selected four areas as shown in Figure 5B to analyze the morphologic features of microglia/macrophages, and selected the contralateral CPu as the control area. In region I, processes of microglia/macrophages were decreased, but still radially distributed (Figure 5C,D). In regions II, III, and IV, the processes were further decreased—especially in region IV, where microglia/macrophages had the fewest processes, ameboid microglia/macrophages even lost their processes (Figure 5C,D). The highest density of microglia/macrophages presented in region III, where they concentrated in arrays and formed a band-like area (Figure 5C,E). In regions II and III, microglia/macrophages had their processes mainly towards the hemorrhagic center, but not radially distributed. To evaluate the directivity of processes, the cell body was set as the origin, and the *y*-axis was set pointing to the hemorrhagic focus (Figure 5F). The directivity was calculated as that of the process numbers in quadrants I and IV deducted the process numbers in quadrants II and III. The difference was divided by the total number of processes. We found that the microglia/macrophages in regions II and III had higher directivity towards the hemorrhagic focus (Figure 5G). Thus, microglia/macrophages in different regions had different morphologic characteristics. In summary, from region I to region IV, the process number of microglia/macrophages decreased successively. Ameboid microglia/macrophages in region IV even had no processes at all. In region I, the processes of microglia/macrophages were radially distributed. In regions II and III, the processes showed obvious directivity towards the hemorrhagic center (Figure 5H). Region III had the largest density of compactly arrayed microglia/macrophages (Figure 5H).

## 3. Discussion

The most frequently used methods to establish animal models of ICH are the intracerebral injections of autologous blood or collagenase in rodents [22,23], and both of them can induce hematomas of various sizes and locations. However, these two methods may bring different pathogenesis of ICH. Autologous blood injection [24] produces a single large bleeding, which is suitable to study the mechanisms of hemorrhage-induced neuronal damage, but fails to reproduce the aspect of continuing bleeding and hematoma expansion. In contrast, collagenase injection [14] destroys the basal lamina of small cerebral blood vessels and results in parenchymal bleeding lasting for some time [22]. In this research, we injected 0.4 U collagenase VII into the left CPu of rats and successfully duplicated an ICH rat model evaluated by behavior tests and MRI tests.

Microglia/macrophages, the first activated innate immune cells in brain, continuously scan the extracellular environment and could be activated quickly after injury [25]. In experimental ICH, microglial activation occurs as early as 1 h following collagenase injection [26] and 4 h after autologous blood injection [27]. Microglia/macrophages elevated to the highest level at 72 h and returns to normal level 3–4 weeks after ICH [28,29]. In this research, both microglia/macrophages and astrocytes were observed in the collagenase VII-injected CPu. However, microglia/macrophages were found much closer than astrocytes to the hemorrhage center by double-label immunofluorescence staining. All of these data indicate the important role of microglia/macrophages as the active sensors and/or cleaners in the hemorrhage foci. Upon stimulation, microglia/macrophages will be rounded gaining an ameboid shape and high phagocytic activity [30]. In this research, we observed multiple shapes of microglia/macrophages in vivo. According to the distance to the hemorrhagic center, we selected four areas—I, II, III, and IV—and found the process numbers of microglia/macrophages decreased successively from region I to region IV. Ameboid microglia/macrophages in region IV even had no processes. The processes of microglia/macrophages were radially distributed in region I; however, they showed obvious directivity towards the hemorrhagic focus in regions II and III. Region III had the largest density of compactly arrayed microglia/macrophages. All these data showed that the activated microglia/macrophages in different regions have different morphologic characteristics, further indicating different functions. For instance, having their processes mainly towards the hemorrhagic center, microglia/macrophages in regions II and III may be migrating to the hemorrhagic center. Compactly arrayed around the hemorrhagic focus, microglia/macrophages in region III may act as guardians to prevent the diffusion of injuries. Ameboid microglia/macrophages in region IV, also Prussian blue positive, may be the effective cleaners of injury.

To analyze the polarization of microglia/macrophages after ICH, we used antibodies specific to CD68 to identify M1, CD206 and Ym1 to identify M2 microglia/macrophages [12,13,17,20] by double-label with Iba-1 or CD11b. By double-label immunofluorescence staining on brain slices, we found ameboid Iba-1/CD11b positive microglia/macrophages were CD68 positive, but neither CD206 nor Ym1 positive. It is difficult to distinguish brain-derived microglia/macrophages from peripheral macrophages that express the same cellular surface markers including CD11b and Iba-1 [31]. In this research, the obvious directivity of microglia/macrophages in regions II and III towards the hemorrhagic center was observed. Thus, we think the Iba-1 positive cells observed in this research were microglia/macrophages. CD68, a lysosomal activity marker expressed by microglia/macrophages in inflammation, is regarded as a marker of M1 microglia/macrophages [18]. In this research, CD68 positive cells in the hemorrhagic center were found to be IL-1β positive, not TNF-α positive. In addition, IL-1β had higher levels at three to seven days after collagenase VII injection. All of this indicates ameboid microglia/macrophages, mainly of the M1 phenotype, in the hemorrhagic foci secreted pro-inflammatory cytokines, selectively.

Beside the clearance of cell debris, microglia/macrophages also play an important role in the phagocytosis of blood components released into the brain [32]. We found most microglia in region IV were Prussian blue positive, indicating that they have strong phagocytosis ability and play important roles in the cleaning of hemorrhages. Therefore, beside blocking the acute detrimental effects of microglia/macrophage activation, stimulating microglial phagocytosis and thus enhancing recovery may also have therapeutic potential.

## 4. Materials and Methods

### 4.1. Antibodies and Chemicals

Collagenase VII and 4’,6-diamidino-2-phenylindole (DAPI) were from Sigma (St. Louis, MO, USA). Collagenase VII was dissolved in physiological saline solution at concentration of 25 U/μL and stored at −20 °C. Before use, collagenase VII was diluted to 0.2 U/μL in 0.9% NaCl. All the antibodies used in this study are listed in Table 1. Prussian blue staining kits were from Leagene (Beijing, China), and Histostain TM-SP kits were from Origene (ZSGB-Bio, Beijing, China).

### 4.2. Animals and Stereotactic Injections of CPus

Three-month-old male SD rats of 250 ± 10 g were supplied by the Experimental Animal Central of Tongji Medical College, Huazhong University of Science and Technology. Rats were kept in cages under a 12 h light/12 h dark cycle with the light on from 7:00 a.m. to 7:00 p.m. All efforts were made to minimize animal suffering and to reduce the number of rats used and all experimental procedures in this research have been approved by the Animal Care and Use Committee of Huazhong University of Science and Technology.

Rats were anesthetized intraperitoneally with 6% chloral hydrate (400 mg/kg) and placed in a stereotaxic frame (Narishige, Tokyo, Japan) at a rat-skull position, with the incisor bar set 2 mm below the ear bars. After the scalp was incised (5–6 mm), the left skull was cleaned and one hole (diameter 1.0 mm) was made for injection at the coordinates AP 0.6, L 3.5, V 5.5 (in mm from bregma and dura, flat skull. AP, anteroposterior. L, mediolateral. V, dorsoventral.) according to the stereotaxic atlas of Paxinos and Watson [33]. A sterilized needle connected a 5 μL syringe was sterotaxically placed into the CPu. The rats were injected with 0.4 U collagenase VII (2 μL, ICH rats) or as a vehicle control, 0.9% NaCl (2 μL, sham rats). The rate of infusion was approximately 0.4 μL/min and the needle was left in place 5 min after injection before being withdrawn. The non-specific pressure effect, which might be caused by infusion rate, was eliminated by vehicle control injection.

### 4.3. Behavior Tests

We performed the beam balance test as reported [34]. Briefly, rats were placed in the middle of a horizontal beam (80 cm of length, 1.5 cm of width), of which one end was fixed on a vertical baffle board and the other end was suspended approximately 50 cm above the floor. When rats were placed on the beam, gesture and time of rats stayed on the beam were recorded. The longest time of each trial was 2 min. A score between 1 (best performance) and 6 (worst performance) was given for each trial, and the side of falling down was recorded too.

The elevated body swing test was first reported in 1995 [35]. In this research, we performed a modified elevated body test. Briefly, the rat was placed on the floor firstly, and then was elevated 10 cm above the floor hanging from its tail. The rat would swing its head and body and try to escape, and then there was an angle between the moved cranioaural axis and vertical axis. When the angle was more than 90°, one time of swing with the swing bias of left or right was recorded. There was a 2 min rest between tests. After 20 times of recording, the rat was put back into the cage. We calculated the percentage of left-biased swings (L) or right-biased swings (R) in the total swings recorded.

### 4.4. Magnetic Resonance Imaging (MRI)

Animal preparation and anesthesia: body temperature of the animal was maintained at 37 °C and the respiration rate was monitored by recording chest wall movements using a piezoelectric device. All the rats were initially anesthetized with isoflurane (2.5% for induction and set-up on the animal bed, 0.8%–1% during experiments) in a 20% oxygen (O_2_)/80% air mixture. MRI experiments were conducted using a Bruker Biospec 70/20USR small animal MR system (Bruker BioSpin MRI, Ettlingen, Germany) operating at 300 MHz (7 T). A four-channel receive array was used in combination with a detunable partial volume transmit coil (Bruker BioSpin MRI). A T2 anatomical reference scan in the coronal plane was acquired using a spin echo (Turbo-RARE (rapid acquisition with relaxation enhancement), sequence: field of view (FOV) = 20 × 20 mm, matrix dimension (MD) = 128 × 128, repetition time (TR) = 2000 ms, echo time (TE) = 10 ms, RARE factor = 4, number of averages (NA) = 8, spatial resolution = 0.15 × 0.15 × 0.6 mm, 12 slices, no gap (containing all the area of cerebral hemorrhages). For magnetic resonance spectral (MRS) analysis in vivo, after local shimming with point resolved spectroscopy (PRESS)-waterline, a single-voxel technique was acquired. Three voxels (core and two borders of cerebral hemorrhages) had spatial resolution equal to 3.5 × 3.5 × 2.4 mm.

### 4.5. Morphological Techniques on Brain Slices

Rats were killed by overdose chloral hydrate (1 g/kg) and transcardially perfused with 500 mL ice-cold 0.9% NaCl followed by 500 mL ice-cold phosphate buffer containing 4% paraformaldehyde. Fixed brains were cut into sections (20 μm) with a freezing microtome (Kryostat 1720, Leitz, Wetzler, Germany) after sinking twice in 25% sucrose. The sections were collected consecutively in PBS for staining.

#### 4.5.1. Immunohistochemical Staining

Free floating sections were blocked with 0.3% H_2_O_2_ in absolute methanol for 30 min and nonspecific sites were blocked with bovine serum albumin (BSA) for 30 min at room temperature then incubated overnight at 4 °C with primary antibodies (Table 1). Immunoreaction was developed using Histostain TM-SP kits (ZSGB-Bio, Beijing, China) and visualized with diaminobenzidine (brown color). For each primary antibody, 3–5 consecutive sections from each brain were used. The images were observed using a microscope (NIKON 90i, Tokyo, Japan).

#### 4.5.2. Double-Label Immunofluorescence Staining

For double-label immunofluorescence staining, free-floating slices were incubated at 4 °C overnight with anti-Iba-1 and anti-GFAP (glial fibrillary acidic protein, Figure 1), anti-CD68 and anti-Iba-1, anti-CD11b and anti-CD206/Ym1 (Figure 3), and anti-IL-1β/TNF-α and anti-CD68 (Figure 4). The immunoreactivity of anti-GFAP was probed using Rhodamine Red-X conjugated goat anti-mouse IgG (H + L) (Figure 1). The immunoreactivity of anti-Iba-1 was probed using Oregon Green 488 conjugated goat anti-rabbit IgG (H + L) in Figure 1 and Rhodamine Red-X conjugated goat anti-rabbit IgG (H + L) in Figure 3. The immunoreactivities of anti-CD11b and anti-CD68 were probed using Oregon Green 488 conjugated goat anti-mouse IgG (H + L) (Figure 3) and anti-CD68 in Figure 4 was probed by Rhodamine Red-X conjugated goat anti-mouse IgG (H + L). The immunoreactivities of anti-CD206 and anti-Ym1 were probed using Rhodamine Red-X conjugated goat anti-rabbit IgG (H + L) (Figure 3). The immunoreactivities of anti-TNF-α and anti-IL-1β were detected using Oregon Green 488-conjugated goat anti-rabbit IgG (H + L) (Figure 4). The images were observed using a laser scanning confocal microscope (Zeiss LSM 710, Oberkochen, Germany).

#### 4.5.3. Prussian Blue Staining

After being washed in double-distilled water (ddH_2_O) three times (5 min/time) and dried in air, brain slices were added Prussian blue staining solution (equal volume of 6% potassium ferrocyanide and 10% hydrochloric acid, temporarily mixed before use) and incubated for 8 h at room temperature. Then, they were stained with 250 μL nuclear red counterstain for 10 min after being washed in ddH_2_O three times (5 min/time). The images were observed using a microscope (NIKON 90i).

### 4.6. Statistical Analysis

Data were analyzed by using SPSS 12.0 statistical software (IBM, Chicago, IL, USA). All data were expressed as means ± standard error of the mean (SEM). In addition, the differences among groups were tested with a one-way analysis of variance (ANOVA) procedure followed by a Student–Newman–Keules test. The level of significance was set at *p* < 0.05.

## 5. Conclusions

As the first activated innate immune cells in brain, microglia/macrophages presented the high morphologic plasticity in the pathogenesis of ICHs. In this research, we observed the increased microglia/macrophages were the nearest cells to the hemorrhagic center after collagenase VII injection into CPu of rats. According to the distance to the hemorrhagic center, we selected four areas, I, II, III, and IV, to analyze the morphology of microglia/macrophages. The processes decreased successively from region I to region IV, where microglia/macrophages were ameboid and had no processes. The processes in region I were radially distributed, however, in regions II and III they showed obvious directivity towards the hemorrhagic center. Region III had the largest density of compactly arrayed microglia/macrophages. The ameboid microglia/macrophages in region IV were Prussian-blue positive, CD68 and IL-1β positive, not TNF-α positive, suggesting their M1 phenotype and strong abilities of phagocytosis and secretion of IL-1β. Thus, activated microglia/macrophages with high morphologic plasticity in the hemorrhagic foci had a strong phagocytosis ability of phagocytosis and secreted pro-inflammatory cytokines selectively.

## Figures and Tables

**Table 1:** Antibodies used in this study.

Antibody	Type	Dilution	Source
Iba-1	Poly-	1:200 for IHC 1:200 for IF	Wako (Osaka, Japan)
CD68	Mono-	1:100 for IF	Abcam (Cambridge, UK)
GFAP	Mono-	1:200 for IHC 1:200 for IF	Abcam
IL-1β	Poly-	1:100 for IHC 1:50 for IF	Santa Cruz Biotechnology (Delaware Ave Santa Cruz, CA, USA)
TNF-α	Poly-	1:50 for IF	Millipore (Billerica, MA, USA)
CD11b	Mono-	1:50 for IF	Bio-Rad (Hercules, CA, USA)
Ym1	Poly-	1:100 for IF	Stem Cell Technologies (Vancouver, BC, Canada)
CD206	Poly-	1:100 for IF	R&D Systems (Minneapolis, MN, USA)
goat anti-mouse IgG (H + L)	Rhodamine Red-X conjugated	1:1000 for IF	Invitrogen (Carlsbad, CA, USA)
goat anti-mouse IgG (H + L)	Oregon Green 488 conjugated	1:1000 for IF	Invitrogen
goat anti-rabbit IgG (H + L)	Rhodamine Red-X conjugated	1:1000 for IF	Invitrogen
goat anti-rabbit IgG (H + L)	Oregon Green 488 conjugated	1:1000 for IF	Invitrogen

Mono-, monoclonal; Poly-, polyclonal; IHC, immunohistochemistry; IF, immunofluorescence.
